# Supplementary figures and images for: Carotid plaque thickness is increased in chronic kidney disease and associated with carotid and coronary calcification
Source: PLoS One. 2021 Nov 23;16(11):e0260417. doi: 10.1371/journal.pone.0260417 (PMC8610240; doi:10.1371/journal.pone.0260417)

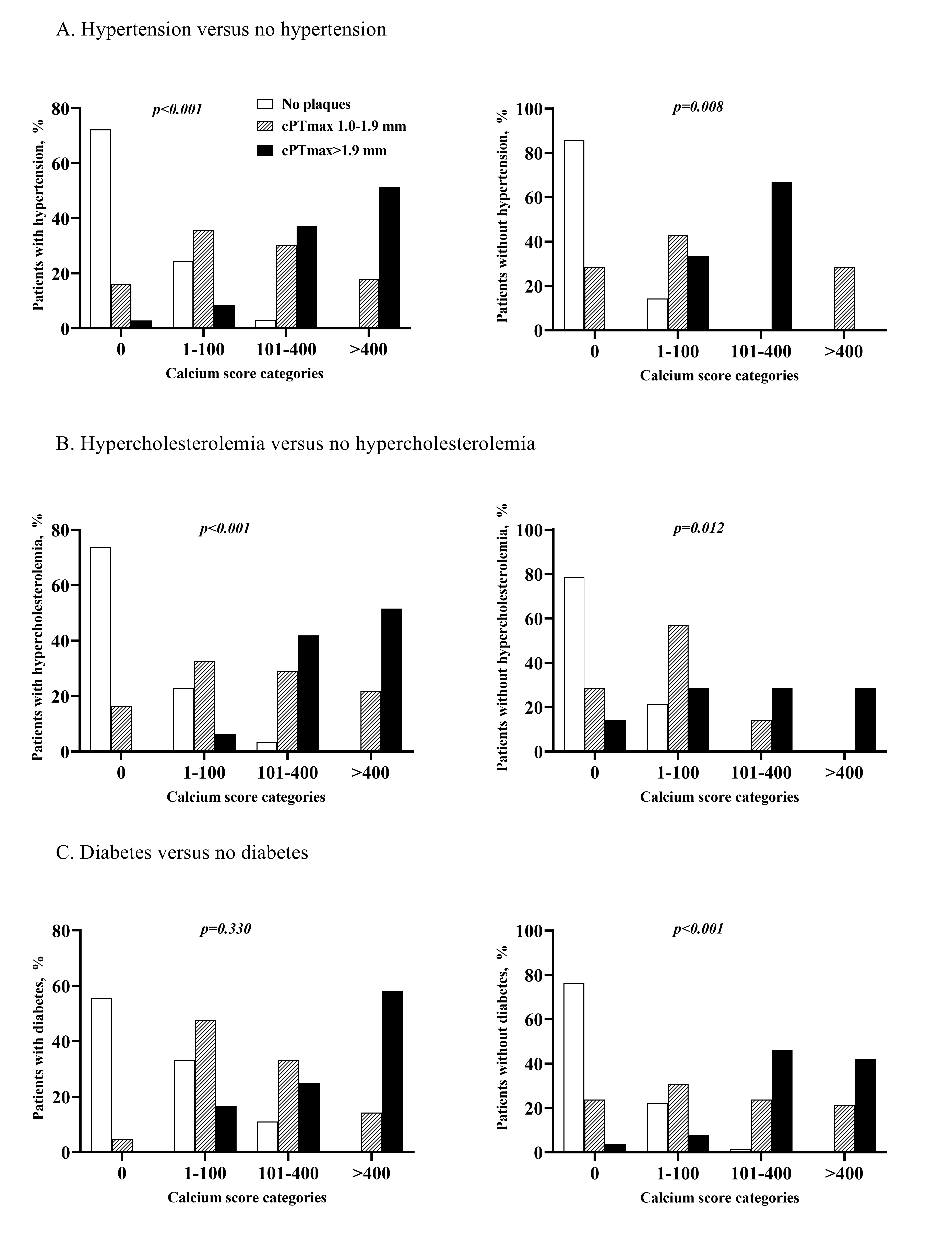

Supplement: S1 Fig — (A) In patients with and without hypertension, (B) in patients with and without hypercholesterolemia, and (C) in patients with and without diabetes. According to carotid ultrasound findings, patients were divided into 3 groups: No carotid plaques, cPTmax 1.0–1.9 mm, cPTmax >1.9 mm. Based on the distribution of calcium scores from CT scanning of the carotid arteries, patients were divided into calcium score categories of 0, 1–100, 101–400 and >400. Hypertension was defined as systolic blood pressure >140 mmHg and/or diastolic blood pressure >90 mmHg or use of oral antihypertensive treatment. Hypercholesterolemia was defined as low-density lipoprotein (LDL) cholesterol >3 mmol/l or treatment with cholesterol-lowering medication. Diabetes: Type 1 or type 2 diabetes. The p-values are from cross tabulation and chi-square analysis (rows: Calcium score categories, columns: cPTmax categories). (TIF) [file pone.0260417.s001.tif]

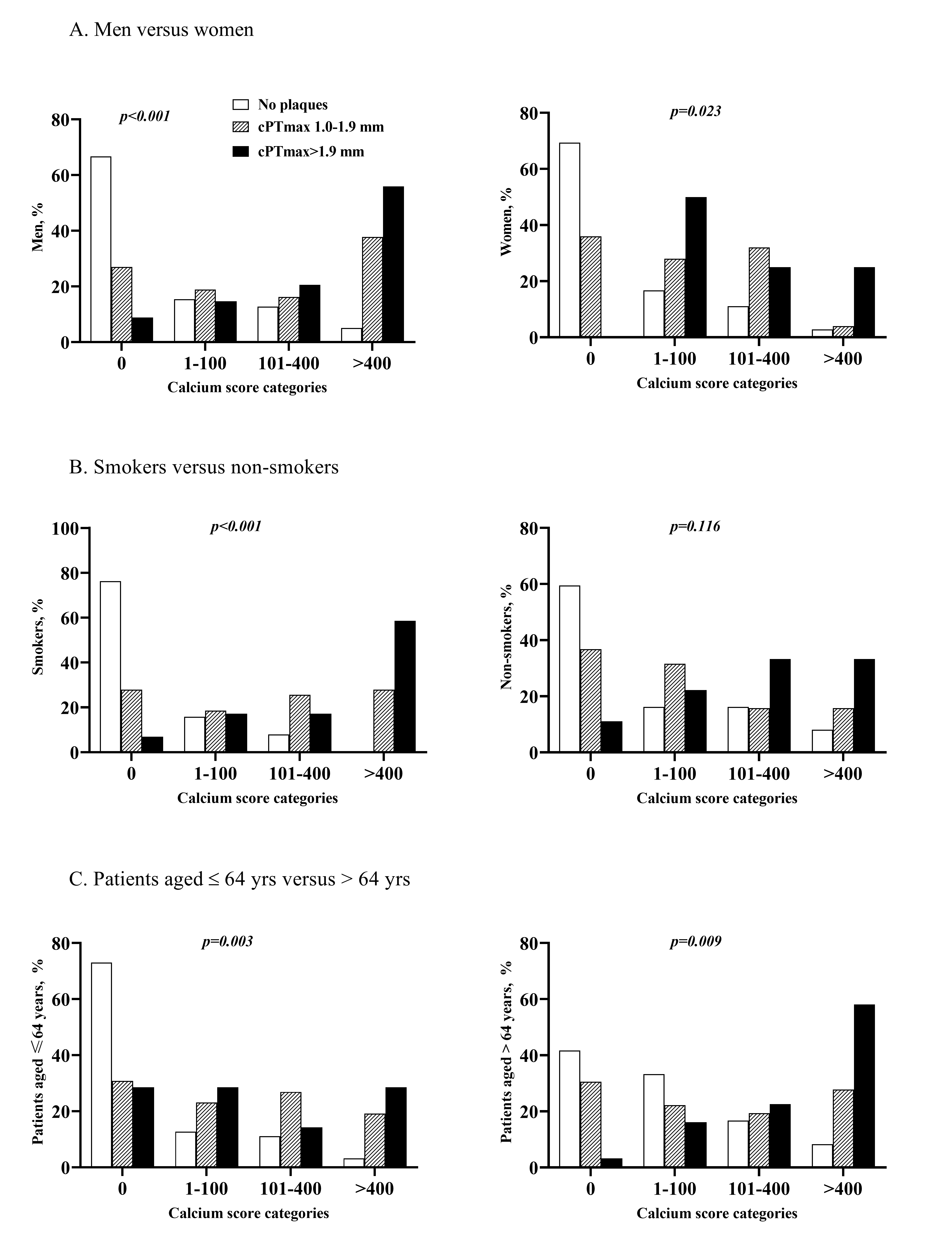

Supplement: S2 Fig — (A) In men versus women, (B) in smokers versus non-smokers, and (C) in patients aged ≤ 64 yrs. versus > 64 yrs. According to carotid ultrasound findings, patients were divided into 3 groups: No carotid plaques, cPTmax 1.0–1.9 mm, cPTmax >1.9 mm. Based on the distribution of calcium scores from CT scanning of the coronary arteries, patients were divided into calcium score categories of 0, 1–100, 101–400 and >400. Non-smokers are defined as patients with 0 smocking pack yrs. and smokers as patients with smoking pack yrs. > 0. Age 64 yrs. is the median age of the CKD patients. The p-values are from cross tabulation and chi-square analysis (rows: Calcium score categories, columns: cPTmax categories). (TIF) [file pone.0260417.s002.tif]

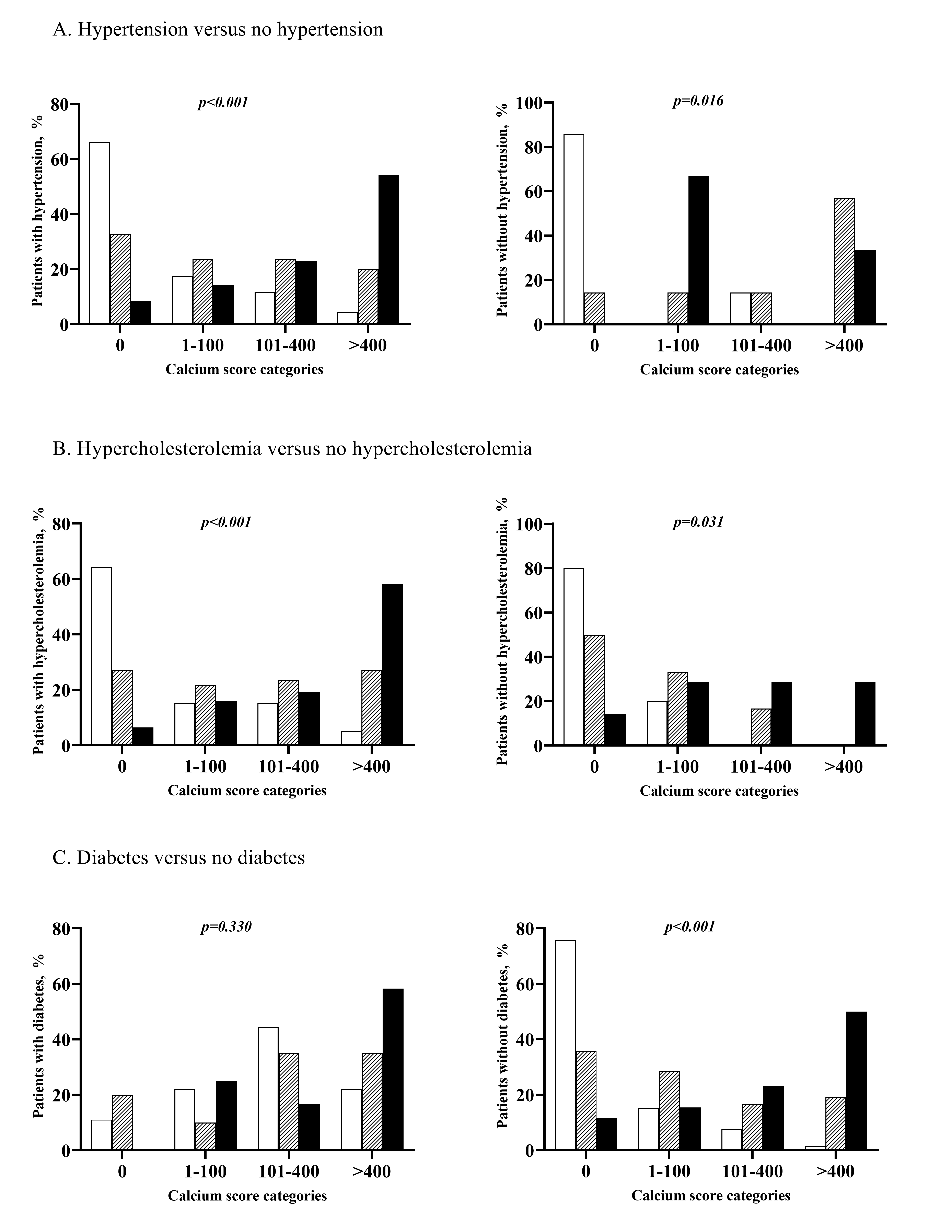

Supplement: S3 Fig — (A) In patients with and without hypertension, (B) in patients with and without hypercholesterolemia, and (C) in patients with and without diabetes. According to carotid ultrasound findings, patients were divided into 3 groups: No carotid plaques, cPTmax 1.0–1.9 mm, cPTmax >1.9 mm. Based on the distribution of calcium scores from CT scanning of the coronary arteries, patients were divided into calcium score categories of 0, 1–100, 101–400 and >400. Hypertension was defined as systolic blood pressure >140 mmHg and/or diastolic blood pressure >90 mmHg or use of oral antihypertensive treatment. Hypercholesterolemia was defined as low-density lipoprotein (LDL) cholesterol >3 mmol/l or treatment with cholesterol-lowering medication. Diabetes: Type 1 or type 2 diabetes. The p-values are from cross tabulation and chi-square analysis (rows: Calcium score categories, columns: cPTmax categories). (TIF) [file pone.0260417.s003.tif]
